# Overview and current management of computerized adaptive testing in licensing/certification examinations

**DOI:** 10.3352/jeehp.2017.14.17

**Published:** 2017-07-26

**Authors:** Dong Gi Seo

**Affiliations:** Department of Psychology, College of Social Science, Hallym University, Chuncheon, Korea; Hallym University, Korea

**Keywords:** Emergency medical technicians, Computers, Licensure, Certification, Psychometrics

## Abstract

Computerized adaptive testing (CAT) has been implemented in high-stakes examinations such as the National Council Licensure Examination-Registered Nurses in the United States since 1994. Subsequently, the National Registry of Emergency Medical Technicians in the United States adopted CAT for certifying emergency medical technicians in 2007. This was done with the goal of introducing the implementation of CAT for medical health licensing examinations. Most implementations of CAT are based on item response theory, which hypothesizes that both the examinee and items have their own characteristics that do not change. There are 5 steps for implementing CAT: first, determining whether the CAT approach is feasible for a given testing program; second, establishing an item bank; third, pretesting, calibrating, and linking item parameters via statistical analysis; fourth, determining the specification for the final CAT related to the 5 components of the CAT algorithm; and finally, deploying the final CAT after specifying all the necessary components. The 5 components of the CAT algorithm are as follows: item bank, starting item, item selection rule, scoring procedure, and termination criterion. CAT management includes content balancing, item analysis, item scoring, standard setting, practice analysis, and item bank updates. Remaining issues include the cost of constructing CAT platforms and deploying the computer technology required to build an item bank. In conclusion, in order to ensure more accurate estimations of examinees’ ability, CAT may be a good option for national licensing examinations. Measurement theory can support its implementation for high-stakes examinations.

## Introduction

The purpose of licensing/certification examinations is to decide whether candidates are qualified to work in a specific professional field. Thus, precisely measuring a candidate’s ability is a major issue in test theories and applications. In order to measure a candidate’s ability exactly, a variety of models and theories have been developed. Classical test theory (CTT) made significant contributions to test development over the course of a long period. However, CTT showed several limitations, and item response theory (IRT) has therefore been applied to measurement theory in licensing/certification examinations since the 1970s. Although conventional tests are based on IRT, a large number of items must be developed to ensure high and equal precision across all candidates. In contrast to a conventional test, computerized adaptive testing (CAT) yields a high and equal degree of precision for all candidates, and requires fewer items than a conventional test to reach a given degree of precision. This paper reviews CAT with respect to measurement theory, presents the components of the CAT algorithm, and explains the aspects of IRT that are required to implement and manage CAT. Specifically, the measurement system for CAT is considered in detail in terms of how to manage a CAT system after implementation, through procedures such as content balancing, item analysis, standard setting, practice analysis, and item development.

## Overview of computerized adaptive testing

### Brief history of computerized adaptive testing

There was a great change in theories of psychological testing during the 1960s. Although CTT had contributed to psychological measurements for a long time [1], it had many limitations in terms of measurements. One of the major assumptions of CTT is the parallel test assumption, according to which true score variance and error variance are the same across 2 tests. However, parallel test forms are impossible in real test situations because the item parameters are dependent on a sample and the person parameters of a sample are dependent on the test. An ideal test model requires invariance properties such as test-independence for different samples and sample-independence for different tests. IRT satisfies the invariance property and has been successful in providing procedures for placing item difficulty parameters and person parameters on an identical scale.

The invariance property of the IRT model makes it theoretically possible to solve some important measurement limitations that were difficult to handle with CTT. One of these problems involves issues of test linking/equating of item parameters for CAT. If a candidate’s ability and the item’s difficulty parameters are placed on an identical scale, equating can be performed without any assumptions about candidate score distributions. This property makes it possible to compare candidates on the same scale even if measurements are made of different groups and with different tests. Since item parameters in IRT are linearly transformable across different samples from a population, the item bank must be large so that all items can be placed on the same scale for CAT [2].

As part of creating measurement instruments, IRT can be used to compute the test information function (TIF), which provides precise procedures to meet specific levels of objectivity. Since the TIF can be obtained by summing the values of item information functions conditional on the candidate’s ability (*θ*), it shows how well a test measures candidates at each value of *θ*. Therefore, the TIF is an index of local precision at the test level and is useful for ensuring desirable exam objectivity and for developing a test instrument that satisfies a target information function. In order to provide each candidate with a precise *θ* estimate, the target information function should be high and constant across *θ*. However, a classical test with a fixed set of items will have low but equal precision across all *θ* [3]. Consequently, for a classical test to have high and equal precision across all examinees, it would require a very large number of items. In contrast to classical tests, CAT can yield a high and equal degree of precision for all candidates, and requires fewer items than a classical test to reach a high level of precision [3].

In many previous studies, it has been repeatedly demonstrated that the number of administered items in CAT is on average 50% shorter than when a paper-pencil test is used, with equal precision [3,4]. Thus, many educational and psychological examinations have been administered using CAT beginning in the 1980s. One of the first large-scale applications of CAT was the College Board’s tests, including reading comprehension, sentence skills, arithmetic, and elementary algebra, which started in 1985. The first application of CAT for a licensing/certification examination was the Novel Corporation’s Certified Network Engineer Exam. This examination was implemented online in 1990 and transitioned to web-based CAT in 1991 [5]. Subsequently, the Graduate Record Examination has been operated by CAT at Sylvan testing centers in the United States since 1992 [6]. Exams for US nurse candidates have been administered using CAT at test centers since 1994 [7]. The Armed Services Vocational Aptitude Battery is administered using CAT at military entrance processing stations [8]. In addition, the Graduate Management Admission Council has provided a CAT version of the GMAT since 1997. The National Registry of Emergency Medical Technicians adopted CAT for emergency medical technician certifications in 2007. Recently, many licensing/certification examinations have been quickly moving to adopt CAT to ensure efficient and accurate measurements. For example, the National Health Personnel Licensing Examination Board in the Republic of Korea is preparing the implementation of CAT for the Korean Medical Licensing Examination [9].

### Item response theory

Most exams using CAT are developed on the basis of IRT. The IRT model was adopted from psychophysics and biology using the item characteristic curve (ICC). Binet and Simon [10] presented plots indicating that as age increased, the probability of a keyed response to an item increased. These plots are referred to as ICCs or item response functions (IRFs). Lawely [11] related IRT perspective parameters to CTT perspective parameters and developed several parameter estimation methods. However, Lawley’s models had limitations due to the assumption of equal item inter-correlations and the absence of a guessing parameter. After that, Lord [12] proposed a more formalized version of IRT models. He developed IRT models associated with the normal ogive models for parameter estimation. Samejima [13] extended the applicability of IRT from dichotomous and unidimensional models to polytomous and multidimensional models.

IRT models can be classified as either dichotomous or polytomous models based on how responses are scored. In dichotomous IRT models, item responses are classified into 2 categories, representing correct (1) or incorrect (0) answers, while they are classified into multiple response categories in polytomous IRT models. Most licensing/certification examinations are based on dichotomous IRT models. There are 2 types of mathematical models in IRT. One is the normal ogive model, which adopts a cumulative normal curve. The other is the logistic model, which is mathematically simpler because a single integral, instead of a double integral as in a normal ogive model, is adopted for examinee trait estimation.

Lord [14] proposed an IRT model in which an IRF takes the form of a normal ogive model. This model was only of theoretical interest before the advent of a new computational technique that was instrumental in avoiding a very complex computation required by the model. An IRT model may use 1, 2, or 3 parameters to define different IRT models. The 3-parameter normal ogive model can be described as:

(1) P(uij=1ai,bi,ci,θj) = ci+(1-ci)∫-∞ai(θj-bi) 12πexp(-z22)dz.

where *P*(*u_ij_* = 1 | *a_i_*, *b_i_*, *c_i_*, *θ_j_*) is the probability of getting an item *i* correct given person parameter and item parameters *θ_j_* is a latent trait parameter (*a_j_*, *b_j_*, ability *c_j_*). *θ_j_* of a person *j*, *b_i_* is the item difficulty parameter for an item *i*, *a_i_* is the item discrimination parameter for an item *i*, *c_i_* is the guessing parameter for an item *i*, and *z* is the standard normal deviate (*a_j_*(*θ_j_*-*b_i_*)). The 2-parameter normal ogive model is a special case of the 3-parameter model, with the *c_i_* parameter removed:

(2) P(uij=1ai,bi,θj) =∫-∞ai(θj-bi) 12πexp(-z22)dz.

The 1-parameter normal ogive model is a special case of the 2-parameter model, taking only the item difficulty parameter into consideration and fixing the *a_i_* parameter at a single value:

(3) P(uij=1bi,θj) =∫-∞(θj-bi)12πexp(-z22)dz.

Birnbaum [15] proposed a IRT model in which an IRF takes the form of a logistic model. The mathematical form of the 3-parameter logistic model (3PLM) is written as:

(4) P(uij=1ai,bi,ci,θj) =ci+1-ci1+exp[-1.7ai(θj-bi]

where *P*(*θ*), *a_i_*, *b_i_*, and *θ_j_* have essentially the same interpretations as in the normal ogive model. The discrepancy in the values of *P*(θ) between the normal ogive models and the logistic models is less than 0.01 for all values of *θ* [16].

The *c* parameter, referred to as the guessing parameter, represents the probability of answering an item correctly regardless of an examinee’s level of *θ*. Thus, an examinee at a very low level of *θ* will have a *c* value as the probability of answering the item *i* correctly. Examinees at a low level of *θ* are affected by the *c* parameter because given difficult items they would randomly guess the correct answer more often than those at a higher level of *θ*. The parameter *b* is usually considered an index of item difficulty. It represents the point on the *θ* scale at which an examinee has a 50% chance of answering the item *i* correctly when *c* is equal to zero [16]. Although the *b* parameter theoretically ranges from −∞ to ∞, *b* values between −2.0 and 2.0 include more than 95% of all cases in the standard normal distribution. Items with values of *b* near −2.0 are very easy items, and those with *b* values near 2.0 are very difficult items. The item discrimination parameter *a* is the slope of *P*(θ) at the point of *θ*=*b*. Although the range of *a* is theoretically from −∞ to ∞, negatively discriminating items are ignored for operational purposes. Thus, the usual *a* value ranges from zero to ∞, with a practical upper limit of about 3.0. A high value of a indicates a steep IRF, whereas a low value indicates a flat IRF.

The 2-parameter logistic model (2PLM) is a special case of the 3PLM where the value of the *c* parameter is zero. The 1-parameter logistic model is, in turn, a special case of the 2PLM where all items have the unit value of *a* and *c* has a value of zero. The Rasch model is the simplest form of the unidimensional IRT model, as discrimination parameters equally anchor 1 as discrimination parameters are equally fixed across all items with the value of 1 across all items [17]. These IRT models have been applied in CAT for several decades.

### Unidimensional computerized adaptive testing

**Fig. 1.**
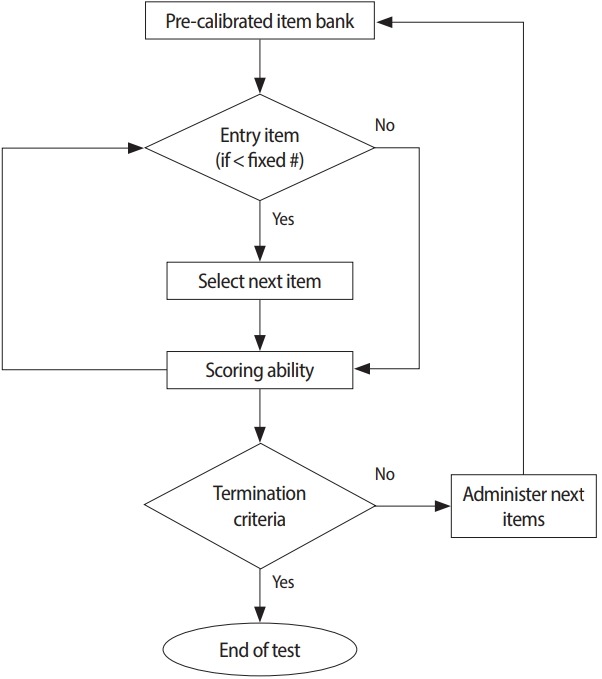
Flowchart of the computerized adaptive testing operational algorithm.

The progress of IRT has enabled powerful quantitative analysis in terms of measurements, such as differential item functioning (DIF), item parameter linking, test score equating, and CAT. During CAT, items are adapted to an individual candidate while he/she is taking an exam. Specifically, CAT allows a test developer to control the exam precision and to maximize the efficiency of the exam. The components of CAT include an item bank, entry point, a procedure for item selection, a scoring method, and the termination criterion of the test. Since the 1970s, research has shown that these components of CAT are most readily achieved by adopting unidimensional IRT [3,4,18]. In line with this development, Thompson and Weiss [3] summarized the 5 steps needed to apply CAT and the operational technique for developing a CAT platform. The first step is to determine whether the CAT approach is feasible for a given testing program. The second step is to establish an item bank. The third step is to pretest, calibrate, and link item parameters via statistical analysis using actual candidates. The fourth step is to determine the specifications of the final CAT related to the 5 components of CAT described in Fig. 1. The fifth step is to deploy the final CAT after specifying all the necessary components. The 5 components of CAT algorithm in licens-ing/certification examinations are briefly explained below.

#### Item bank

A prerequisite for implementing CAT is developing a large bank with many items. A bank may contain over thousands of items, and all items are assumed to measure a single ability on the same scale. It is very difficult to gather a single group of thousands of subjects to develop a large item bank with many items. Therefore, it is required to link subsets of items administered to different groups onto a reference group to create a large item bank. IRT offers pre-calibrated item parameters and a reasonable method for linking exam items due to the invariance properties of parameters for items and candidates. As a result, linking procedures in IRT enable an item bank to have over thousands of pre-calibrated items prior to implementing CAT [19].

#### Starting item

A starting item should be determined before implementing CAT. Usually, the choice of a starting item in CAT is arbitrary because it is difficult to obtain valid prior information about the ability level of a candidate. In theory, selecting the difficulty level of a starting item to be close to the candidate’s ability level improves the efficiency of CAT [20]. In reality, since CAT begins with an item difficulty level of 0, such an item would be readily overexposed. Therefore, several possible methods have been proposed to reduce the item exposure rate. One possible method is to use random selection of the first few items from a subset of the item bank. One specific procedure for determining a starting item in CAT is to combine IRT and Bayesian statistical methods [21,22] and to use external factors to estimate candidate ability [23].

#### Item selection rule

The most important component of CAT is the item selection rule, which continues the CAT procedure after assigning a starting item for a candidate. Item selection rules in CAT are based on the item information function in IRT. Given the current estimate of a candidate’s ability, the most informative item among the remaining items should be chosen for the next item. By using computer software, the maximum information procedure and Bayesian selection procedures are available for item selection. The maximum information procedure allows a CAT to select an item with the maximum information at the candidate’s current ability level. Bayesian selection is used to select the item minimizing the expected posterior variance of the ability estimates [24]. If the purpose of the exam is to classify candidates based on a cut-off score, a likelihood ratio approach is more efficient to use as the item selection rule [25].

#### Scoring procedure

Updating a candidate’s ability level can be performed after each item is administered in a CAT, and the next item to be administered can be selected based on both the candidate’s ability level and all responded items. A candidate’s ability level can be estimated by maximum likelihood or Bayesian methods [22]. If item parameters are assumed to be known, the candidate’s ability level can be estimated from the likelihood function, which is the product of all IRFs. Usually, the local maximum value of the likelihood function given an ability level can be obtained by setting the first derivative of the natural log of the likelihood function at zero. However, maximum likelihood methods can be used only when there is a mixed response pattern. In contrast, Bayesian methods can be used for any response pattern because they are based on Bayes’ rule, which is proportional to the product of the maximum likelihood and prior probability, which usually assumes a standard normal distribution. In Bayesian estimation methods, the Bayesian type estimator is used to find the maximum value of a posterior distribution of ability. The expected a posteriori method is used to find the mean of the posterior distribution of ability.

#### Termination criterion

The final component of CAT is a termination criterion to determine when a candidate stops the exam with a pre-specified degree of precision. The choice of the stopping rule in CAT can vary depending on the purpose of CAT. One criterion is to use the standard error of measurement, which allows a CAT to terminate when the standard error of ability estimates reaches a pre-specified value. The other is the variance of posterior distribution in Bayesian ability estimation methods, according to which CAT terminates when the variance of the posterior distribution becomes smaller than a pre-specified value. In many licensing exams, a CAT is terminated when a candidate is determined as passing or failing based on a cut-off score. CAT is continued until either the candidate’ ability estimate confidence interval is significantly above or below the cut-off score or the candidate completes the maximum number of items. If a candidate completes the maximum number of scored items, the pass/fail decision is determined by ignoring the confidence interval. For these candidates, if the final ability estimate is above the cut-off score, the candidate is given a pass decision; otherwise, the candidate is given a fail decision.

**Fig. 2.**
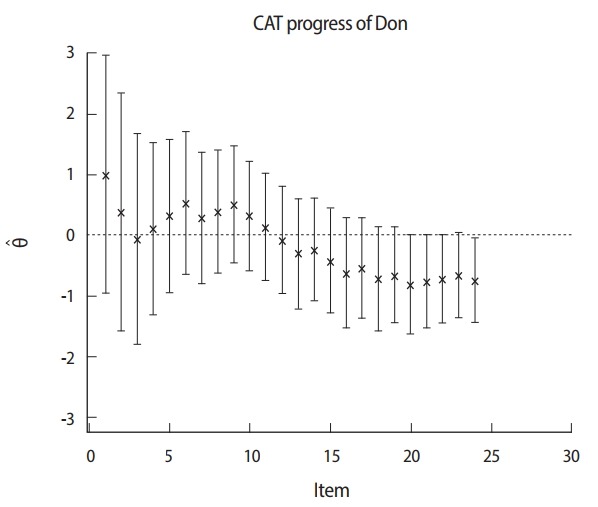
CAT termination using the confidence interval of the ability of a non-master candidate. CAT, computerized adaptive testing.

**Fig. 3.**
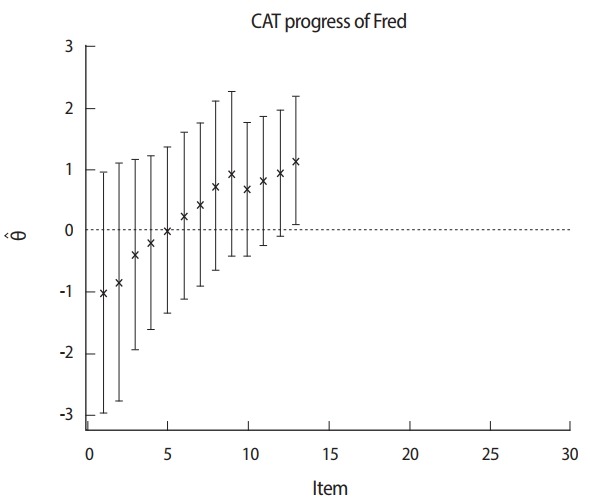
CAT termination using the confidence interval of the ability of a master candidate. CAT, computerized adaptive testing.

Fig. 2 shows that the provisional ability and termination point of the CAT procedure are associated with standard errors for a candidate taking a 24-item CAT. The ability scale is shown on the vertical axis (−3.0 to 3.0). The sequence of 24 adaptively administered items is shown on the horizontal axis. Initially, a candidate starts with a starting item with an item difficulty parameter near zero. After the first item is given, the estimated ability immediately begins to increase because the candidate responds correctly. The range of each error band indicates the relative amount of error associated with the ability estimates. Wider bands mean more standard error, while narrow bands mean a small standard error. If more items are administered, the error bands rapidly narrow. Over 20 items, the estimates for this candidate gradually converge to an ability level of around −1.0. CAT is terminated after 24 items, and the candidate is failed because the upper bound of the confidence interval is below the cut-off score, which is taken as zero in this example. In contrast, Fig. 3 shows an example in which CAT is rapidly terminated at 13 items, and the candidate is passed because the lower bound of the confidence interval is above the cut-off score of zero.

### Computerized adaptive testing management

CAT management ensures that all exam specifications are correctly implemented. CAT management is performed after the deployment of each exam based on CAT through the collaboration of the test developers, a quality check committee, and a psychometrician. Specifically, CAT management includes content balancing, item analyses/scoring, standard setting, practice analysis, and item bank updates. This management plan follows a similar structure for all licensing/certification examinations.

### Content balancing

Content balancing is the most important part of CAT management for licensing/certification examinations. Much of the research into CAT, and most applications of CAT, have been in the context of licensing/certification exams. Abilities in licensing/certification exams are mostly considered both unidimensional and relatively homogenous. Some licensing/certification exams may measure a single component, while others may measure a relatively unidimensional domain, with 2 or more content domains underlying the primary dimension. However, unidimensional CAT does not consider the varied content categories of the items within an item bank as part of the statistical item selection procedure. In order to consider several homogeneous scales in a CAT setting, various procedures have been proposed to achieve “content balance” among candidates in several domains [26,27].

Kingsbury and Zara [27] proposed an algorithm to control content on an item-by-item basis as items are administered. Contentbalanced CAT provides candidates with a test that adequately represents each of the content domains included. For example, content-balanced CAT would administer items according to a pre-specified ratio of content, such as 50% from math content and 50% from verbal content. However, by modifying the maximum information item selection procedure, content balancing would decrease the efficiency of CAT, which would in turn result in longer tests than a pure CAT to reach the test objective (assuming that test length is allowed to vary). In addition, in order to balance the content domains, the percentage of items being administered should be calculated for all content domains before implementing CAT, meaning that practice analysis is required. Furthermore, the content balancing procedure does not provide a candidate with an estimated ability level in each content domain, but with only a single estimate of general ability based on the test [28].

One application of content balancing procedures in CAT is to measure a candidate on multiple scales. McDonald [29] confined homogeneity to unidimensionality in order to extract distinct scales from hundreds of items. If researchers consider each homogeneous scale as a single unidimensional scale, a CAT with multiple scales can not only achieve content balancing for CAT, but also take into account multidimensionality in latent abilities. However, since a CAT with multiple scales proceeds separately for each scale to measure each candidate, it does not consider correlation among abilities. Even though a CAT with multiple scales provides each candidate with an ability score for each content scale, it is not a practical procedure because most test batteries with multiple scales result in scores that are intercorrelated to some degree. Each ability score is usually correlated across different scales, with reported correlations ranging from r= 0.30 to r= 0.50. Therefore, Brown and Weiss [30] tried to consider the ability score correlations across different scales in CAT. Different starting values were generated in reference to inter-scale correlations that were obtained using ability estimates of a test development group.

Another method for balancing content in CAT is to use computerized multistage testing (MST). MST is similar to CAT in that the candidate responds to items determined on the basis of previous item responses. Unlike CAT, MST includes several item sets across modules. A candidate’s ability is estimated by adaptive modules, which guarantees high and equal precision. One of the advantages of MST is to allow candidates to review items within each module. However, MST is dependent on the decisions made about each module by test developers [31].

The most complicated method for controlling content is to use multidimensional CAT instead of unidimensional CAT. Initially, Segall [32] applied multidimensional Bayesian item selection and scoring procedures to CAT, and demonstrated that the resulting multidimensional CAT was more efficient than unidimensional CAT in terms of efficiency and accuracy. In addition to its improvements in efficiency, multidimensional CAT can be used as an instrument for balancing different content domains for candidates [17]. Luecht [33] also demonstrated that multidimensional CAT with content balancing can achieve approximately the same precision with 25% to 40% fewer items than were required in unidimensional CAT with respect to measurement of ability.

### Item analyses

In general, a test publication window should be defined for licensing/certification examinations. For example, several test windows may exist per year to maintain test security. All licensing/certification exam items are analyzed at various stages during the test window for different purposes. Preliminary item analysis (PIA) for operational items is performed after several months of test administration. The purpose of PIA is to evaluate the operational items/test statistics and to identify possible issues with operational items at an early stage of testing. Pretest-item calibration is performed after the test window closes. The purpose of pretest-item calibration is to estimate the difficulty and evaluate the quality of newly written items, and to provide suggestions about future application of these items. Test monitoring is also performed at the end of the test window. The purpose of test monitoring is to examine the stability of the operational-item parameter and to adjust it as necessary. The details of each item-analysis system are presented below.

#### Preliminary item analysis

PIA is conducted to identify any potentially problematic items (e.g., miskeys). Only operational items are considered in the PIA. Usually, the item/test report will be created as a test result of PIA. The item report contains item data and statistics including item ID, number of items, item mean, item total correlation, and descriptive statistic for response time (mean, standard deviation, median, minimum, and maximum). The test report includes the raw scores of candidates (the number of candidates, mean scores, minimum, maximum, and standard deviation), total response time of candidates (mean, total response time, minimum, maximum, and standard deviation), and the licensing/certification pass rate of candidates (frequency and percentage). The item and the test reports are published and are considered for the next CAT window [34].

#### Pretest-item calibration

Pretest-item calibration includes estimating item parameters (discrimination and difficulty) and evaluating item quality [34]. A sample for pretest-item calibration should be defined for professional licensing/certification examinations. Additional rules can be applied to pretest-item calibration sampling depending on the licensing/certification examination. Unusual candidates are excluded from the calibration sample. Pretest items are calibrated based on the IRT model using statistical software (Winsteps, IRTPRO, or R). The scored items (operational items) serve as the anchor to link the current scale to the previous cycle. Pretest items are evaluated and classified based on the following pretest item screening rules: (1) option analysis (percent for each option, item mean, item-total correlation, potential miskey), (2) DIF analysis (Mantel-Haenszel delta and standard mean difference), (3) IRT parameters (difficulty parameter and discrimination parameter), and (4) fit statistics (residual between real data and the theoretical model). Usually, several rules are applied together to screen flagged items. For example, if the item-total correlation is above 0.3, the item discrimination parameter is larger than 1.0, and the difficulty parameter ranges from −2 to 2 without any DIF, the item will be approved for operational items. Otherwise, if the item-total correlation is less than 0.3, the item discrimination parameter is less than 1.0, or the item difficulty parameter is less than −2 or larger than 2, the item will be re-piloted for the next window or deleted [35].

#### Test/item monitoring

After each operational test pool is rotated out of the field, psychometric analyses are conducted on the items to determine their ongoing performance. The possibility of changes in item statistics should be considered for each item that has been administered to several hundred candidates. When significant item difficulty parameter drift appears in an operational item, the item difficulty may require an adjustment to better reflect its current difficulty [36]. Test/item monitoring is performed along with item calibration to evaluate the degree of item parameter drift. Operational item parameter drift should be evaluated when operational items are estimated by several hundred candidates. Usually, items that meet the sample size requirement are estimated and treated as follows. Any items yielding moderate change in the same direction in 2 successive operational pools will be adjusted as the average of the 2 item difficulty parameters in each pool. Items showing large changes will be immediately removed from operational use for the next exam window [35].

### Standard setting

For licensing/certification examinations, the cut-off score should be pre-determined before CAT administration. Standard setting refers to the process of determining the cut-off score for licensing/certification examinations [37]. The main issue in standard setting is the need to have a detailed discussion of the practice of licensing/certification examinations and minimal competence as it relates to entry-level practice. A discussion about the practice of licensing/certification examinations can be initiated by having the participants review the general definition of minimal competency for licensing/certification examinations [37]. The first step of standard setting is to define the borderline minimally competent candidate. Following a review of the general definition of minimal competency, the panel is asked to engage in a more detailed discussion of minimal competency. For each content domain, the panel group will create a list of the knowledge, skill, and ability (KSA) of a borderline minimally competent candidate. After discussing the characteristics of the borderline minimally competent candidate, the panel is trained on the standard setting procedure. In order to rate an item, the panel is asked to estimate the percentage of borderline minimally competent candidates who would answer that item correctly, based on the discussion of borderline minimally competent candidates and the content of the item [38]. For each item, the panel is instructed to ask themselves, “How many borderline minimally competent exam candidates, out of 100, would answer this item correctly?” After the initial round of individual ratings, the panel arrives at a decision regarding the item’s rates. During the group discussion, the panel is provided a feedback summary showing the minimum, maximum, and mean rating for each item. The panel members are encouraged to provide a rationale for their ratings with the highest or lowest ratings for each item. The entire panel discusses items that have a wide range of ratings, or items with a large difference between the mean ratings and item mean statistics. During the discussion, the panel is advised to focus on how a borderline minimally competent candidate would perform on these items. Following the group discussion of each item, the panel members are instructed to reconsider their own rating of the item using any new information and feedback regarding item data. After re-consideration of the first rating, the panel is instructed to give a second or “final” rating for each item. Final ratings are determined as the final estimates of the ratings and are used to calculate the passing cutoff score [37].

### Practice analysis

The purpose of licensing/certification examinations is to ensure that candidates who practice an occupation have met certain standards (American Educational Research Association) [39]. The standard of licensing/certification examinations is usually that a candidate is qualified to practice a particular job. To meet this purpose, licensing/certification examination must include content and tasks reflecting KSA about the profession. This requirement of KSA is typically ensured by developing test plans based on a job analysis. Specifically, practice analysis focuses on the practice-related information contained in job analyses [40]. Practice analyses require information from practitioners, supervisors, and educators working in that field. The practice analysis committee establishes the test blueprint for each content area of licensing/certification exams. Licensing/certification exams must be updated in light of the results of a formal practice analysis study at least once every 5 years [34]. Practice analysis committees are composed of a representative sample of individuals with appropriate experience. Licensing/certification examinations staff must consistently recruit and select subject matter experts for the practice analysis committees. Practice analysis committees adopt several methods to conduct the practice analysis based on the recommendations of practitioners. This methodology must include a review of the exam literature and changes in the relevant scopes of practice, a qualitative sample group, and a validation survey. Practice analysis committees review the results of the practice analysis and propose test blueprints for examinations based on the results of the practice analysis. Statistical researchers produce a formal practice analysis report documenting the methodology, results, and conclusions of the practice analysis and distribute the test blueprint and the practice analysis report publicly [34].

### Item bank updates

New items for licensing/certification examinations are written each year to maintain the item banks [41]. The number of items and their content are determined by psychometricians based on the outcomes of an empirical gap analysis (gap between number of items in the real item bank and the theoretical item bank). New items can be incorporated into the item bank using a linking method. IRT provides a unique method of linking subsets of test items due to the invariance property of the item parameters, which means that the sample ability is independent of item parameters and item parameters are independent of the sample ability within a linear transformation. Several linking methods have been suggested in previous research. The mean/mean method uses the mean of the *a*-parameter estimates for the slope of the linear transformation, and the mean of the *b*-parameter estimates for the intercept of the linear transformation [42]. The mean/sigma method uses the means and standard deviations of the *a*- and *b*-parameter estimates from the common items [43]. The ICC method finds the linking coefficient by using the sum of the squared differences between the ICCs for each item given a particular ability [44]. The test characteristic curve method uses the squared difference between the test characteristic curves for a given ability [45]. Before new licensing/certification exam items are administered as operational test items, they must undergo a process in which the item is administered as a pilot item for the purpose of collecting item information. A pretest pool is constructed and published with each version of the exam. The number of items in the pretest pool is determined by test development experts and psychometricians. The items for the pretest pools will be selected from the group of items that have been approved for pretest use. In CAT administration, pretest items are selected at random from the pool of pretest items. Each pretest item must be administered to a sufficiently representative sample in order to collect information on the performance of the item. Pretest items are incorporated into the operational test so that candidates cannot recognize the difference between operational and pretest items. In order to reduce the effects of warm-up or fatigue, pretest items are not administered at the beginning or the end of CAT. Pretest items are administered after several operational items are assigned. Finally, pretest items are selected as operational items in a CAT item bank through pretest item calibration [34].

## Conclusion

Over the course of several decades, research has repeatedly demonstrated that CAT is more efficient than paper-and-pencil tests, with equal or better measurement precision [3,4]. This review underscores the fact that CAT fosters better licensing/certification examinations than conventional tests based on CTT and IRT, and describes the fundamental components of implementing CAT for individual candidates. Specifically, this review addressed several procedures regarding CAT management after CAT is implemented for licensing examinations. CAT management was defined in terms of several procedures, including content balancing, item analyses, standard settings, practice analysis, and item bank updates. Individual procedures are also distinct research areas in measurement theory. Thus, more details about each procedure in CAT are beyond the scope of this review and will be addressed in future research. Doubtlessly, additional practical issues have been left out of this review of CAT management, such as costs and computer technology. This review has attempted to present an overview of CAT and to consider the key procedures related to operational CAT management. Further issues, such as CAT simulation studies, the costs of constructing CAT platforms, and the computer technology required to build an item bank for a live CAT should be considered. Clearly, further operational issues about CAT should be considered after implementing CAT for licensing/certification examinations because the purpose of a professional exam can be accomplished through consistent management of CAT.

## Supplementary materials

Supplement 1. Audio recording of the abstract.
